# Pharmacological Agents for Soft Tissue and Articular Injections: From Mechanisms to Clinical Applications

**DOI:** 10.3390/jcm15031227

**Published:** 2026-02-04

**Authors:** Jeries Issa Alghishan, Bogdan Andor

**Affiliations:** 1 Department of Orthopedics and Traumatology, Victor Babeş University of Medicine and Pharmacy, 300723 Timişoara, Romania; 2 Teodor Şora Research Center, Victor Babeş University of Medicine and Pharmacy, 300723 Timişoara, Romania

**Keywords:** intra-articular injections, soft tissue injections, corticosteroids, hyaluronic acid, platelet-rich plasma, local anesthetics, osteoarthritis, tendinopathy, regenerative therapies

## Abstract

Soft tissue and intra-articular injections are common treatments for musculoskeletal disorders, especially chronic tendinopathies and osteoarthritis. In this structured narrative review, receptor-level molecular mechanisms are combined with clinical evidence from various studies for the most common injectable treatments, such as corticosteroids, hyaluronic acid, platelet-rich plasma, and local anesthetics. When combining mechanistic pathways with the results of randomized controlled trials, systematic reviews, and network meta-analyses, clear differences between injectable drugs regarding rapidity of action, duration of action, systemic effects, and safety can be appreciated. Corticosteroids alleviate symptoms rapidly but with only short-term duration. Hyaluronic acid exerts longer-lasting biomechanical and anti-inflammatory effects. Platelet-rich plasma showed the most consistent long-term effect on function and regeneration. Local anesthetics remain useful for diagnostic and interventional purposes but prove harmful to cartilage after excessive administration. A mechanism- and phenotype-based framework for selecting injectables is hereby proposed, wherein treatment could be more individualized and clinical decisions could be optimized in musculoskeletal care.

## 1. Introduction

Recent studies increasingly stress the need for phenotype-driven selection of injectable therapies in musculoskeletal disorders. Multiple studies, including those by Pereira et al. [1] and Jones et al. [2], show that osteoarthritis (OA) phenotypes that are predominantly inflammatory respond better to corticosteroid injections, whereas structurally degenerative phenotypes respond more favorably and durably to platelet-rich plasma (PRP) or hyaluronic acid (HA). Large-scale meta-analyses conducted by Bensa et al. and Gupta et al. further support this concept, showing that biologic injectables generally outperform corticosteroids (CSs) in long-term outcomes. These findings indicate that uniform treatment strategies are suboptimal and that aligning therapy with underlying biological mechanisms improves clinical results [1,2,3,4,5,6]. Such injectable treatments are mainly employed for the treatment of adult patients with conditions of knee osteoarthritis, chronic tendinopathy, and periarticular inflammatory disorders, where the treatments are either ineffective or where the specific targeting of biological agents is warranted.

In the last ten years, the use of soft tissue and intra-articular injections for therapy has changed a lot. We now know more about inflammatory and degenerative molecular pathways, biologic processing techniques have improved, and there are more high-quality randomized controlled trials. All of these things have changed how we practice medicine. Recent network meta-analyses show that CSs, HA, and PRP are the best injectable treatments for knee osteoarthritis and other musculoskeletal problems. Current clinical guidelines stress how important it is to make treatment plans that are specific to each patient. For instance, they say to use CSs for sudden flare-ups of inflammation, HA for joint changes that get worse over time, and PRP for structural problems that last a long time.

There are a lot of things that happen at the molecular level that cause musculoskeletal degeneration. These include pro-inflammatory cytokines (like interleukin-1β and tumor necrosis factor-α), more active matrix metalloproteinases, oxidative stress, and problems with mitochondria. This causes cartilage, tendons, and other tissues around joints to slowly break down. CSs alter glucocorticoid receptor signaling; HA interacts with CD44 and RHAMM receptors; PRP activates anabolic growth factor–mediated pathways that are controlled by growth factors; and local anesthetics (LAs) block voltage-gated sodium channels.

The biological and clinical actions of HA are dependent on its molecular weight (MW) and formulation. Low-molecular-weight HA (LMW-HA) primarily induces anti-inflammatory actions through toll-like receptors and transient engagement of CD44, whereas high-molecular-weight HA (HMW-HA) demonstrates improved viscoelastic and chondroprotective properties with longer intra-articular duration via sustained CD44 and RHAMM engagement. Regarding durability, reticular or cross-linked forms of HA have been shown to improve their resistance to enzymatic breakdown, displaying longer durability and mechanical stability with respect to their linear analogue. More recent analysis of the pharmacology of cross-linked HA suggests improved duration of effect with compromised sensitivity of patients during its prolonged use, including those characteristics associated with stiffness [6,7].

Recent reviews published in *Nature Reviews Rheumatology*, *Cartilage*, and *Radiology* emphasize that mechanistic stratification and biomarker-based prediction models should be integrated to guide injectable therapy selection [2,5,8].

This review’s goal is to combine molecular mechanisms and clinical evidence to support injectable strategies in musculoskeletal care that are based on mechanisms and phenotypes. This review seeks to integrate the molecular mechanisms and clinical evidence of frequently utilized soft tissue and intra-articular injectables to facilitate a mechanism-guided, phenotype-driven methodology for treatment selection.

The manuscript is intentionally framed as a structured narrative review with analytical synthesis rather than an experimental or meta-analytic study.

### 1.1. Search Strategy and Sources of Information

The aim of this manuscript was to create a structured narrative review that includes aspects of systematic methodology, while omitting quantitative meta-analysis. A comprehensive literature review was conducted to identify evidence regarding pharmacological agents utilized for soft tissue and intra-articular injections, including CSs, HA, PRP, and LAs. This manuscript is a structured narrative review that includes parts of a systematic review but is not a quantitative meta-analysis.

We searched electronic databases like PubMed/MEDLINE, Scopus, and Web of Science for articles that were published between January 2010 and January 2025. Search strategies used both free-text keywords and Medical Subject Headings (MeSH). These included words like “intra-articular injection,” “soft tissue injection,” “corticosteroid,” “glucocorticoid,” “hyaluronic acid,” “viscosupplementation,” “platelet-rich plasma,” “local anesthetic,” “lidocaine,” “bupivacaine,” “knee osteoarthritis,” “tendinopathy,” and “musculoskeletal disorders.” We used Boolean operators like AND and OR to narrow down our searches. We went through reference lists of important reviews and meta-analyses by hand to find more studies that were relevant.

### 1.2. Eligibility Criteria

Studies were included if they met the following criteria:

1. Peer-reviewed publications in English.
2. Adult human populations (≥18 years).
3. Randomized controlled trials, systematic reviews, meta-analyses, narrative reviews, mechanistic studies, or clinical guidelines.
4. Evaluation of intra-articular or soft tissue injections of CSs, HA, PRP, or LAs.
5. Reporting of clinical outcomes, safety data, mechanistic insights, or comparative effectiveness.

Exclusion criteria included animal-only or in vitro studies without clinical relevance, non-injectable interventions, surgical or non-pharmacological treatments, case reports, conference abstracts, editorials, letters, and non-English publications. The outcomes of interest included clinical effectiveness, duration of therapeutic effect, safety and adverse events, and relevance to underlying pharmacological and molecular mechanisms.

### 1.3. Study Selection

**Figure 1:**
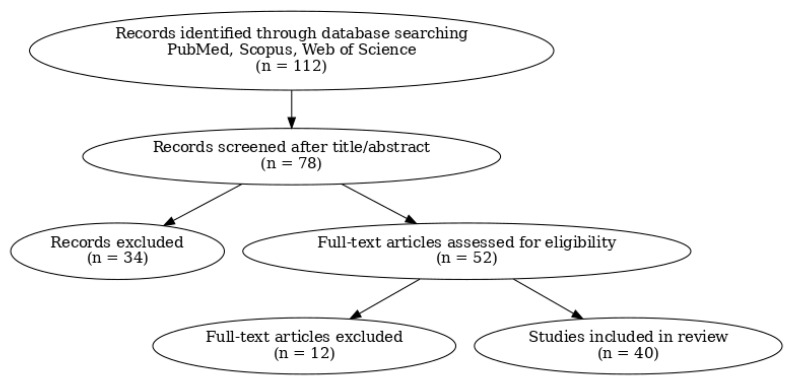
Study selection flow diagram.

Study selection was performed in stages. Titles and abstracts were initially screened to exclude irrelevant records. Full-text articles were then assessed against predefined inclusion and exclusion criteria. Following full-text evaluation, 40 studies were included in the final synthesis and formed the evidentiary basis for mechanistic and clinical analyses. The study selection process is summarized in Figure 1. The study selection diagram (Figure 1) provides a quick overview of how the studies were chosen.

Generative AI tools (GPT-4, version 4.0) were used to edit the language and make the manuscript easier to understand and read. The authors read through the manuscript, made changes, and took full responsibility for what it said.

### 1.4. Pathophysiology and Molecular Targets

Matrix biomechanics are crucial for degeneration, not just the usual inflammatory mediators. Ferkel et al. [9] reported that reductions in the molecular weight of HA compromise joint shock absorption and lubrication, accelerating cartilage degeneration. Studies on PRP also support this. Through the PI3K-Akt pathway, growth factor-rich plasma boosts anabolic signals and slows down cell death. T2 mapping can identify early signs of cartilage dehydration and subtle structural changes on imaging [8]. This type of imaging can help figure out who will really benefit from HA or other joint injections. There is also clinical evidence that HA’s properties lead to better results.

Degeneration is a complicated mix of biochemical events that happen in the synovial fluid, cartilage, and connective tissues around tendons. Chronic inflammation increases the transcription of NF-κB and AP-1, which in turn increases the levels of IL-1β, TNF-α, COX-2, nitric oxide synthase, and MMP-13. All of these things break down cartilage and change the structure of tendons. Their problems with mitochondria and oxidative stress do not go away, and when macrophages become more inflammatory, the breakdown of local tissue speeds up even more.

T2 mapping, dGEMRIC, and synovial diffusion measures are among the most robust imaging biomarkers correlating with structural and molecular changes in osteoarthritis [8].

They can even help predict who will respond to injection treatments.

All these insights lead to more accurate treatments:

- Corticosteroids (CS) quickly lower levels of prostaglandins and leukotrienes by shutting down the NF-κB pathway and phospholipase A2.
- HA connects with CD44 and other receptors to slow down the activity of inflammatory genes, stabilize the matrix, and restore the natural springiness of the joint.
  - PRP is full of growth factors like PDGF, VEGF, IGF-1, and TGF-β that start new blood vessel growth, build collagen, and stop catabolic signals.
  - Local anesthetics (LAs) do not just numb by blocking sodium channels; they also affect mitochondria and turn on proteases, which makes high doses harmful to cartilage.

These pathways are all mixed up, so matching therapy to mechanism is not just a beneficial idea; it is the basis of modern, precise care for osteoarthritis and other musculoskeletal diseases.

CS exert their effects primarily through activation of the glucocorticoid receptor, leading to inhibition of NF-κB-mediated inflammatory signaling and downstream suppression of prostaglandin and cytokine synthesis. CSs inhibit inflammatory signaling through glucocorticoid receptor activation, HA reinstates viscoelasticity and regulates inflammation via CD44-mediated pathways, PRP facilitates tissue repair through growth factor signaling, and LAs obstruct voltage-gated sodium channels while demonstrating dose-dependent cytotoxicity.

### 1.5. Role of Corticosteroids (CSs) in Musculoskeletal Treatments

As mentioned in the previous subsection, CSs are very effective at reducing inflammation because they stop inflammatory signaling pathways from working through glucocorticoid receptors.

Many high-quality studies have indicated that CSs work well for short-term symptom flare-ups, but their long-term effects on structure are still a cause for concern. Many systematic reviews and meta-analyses show that intra-articular corticosteroid injections can temporarily improve pain and function in a clinically significant way [10,11,12,13]. Glucocorticoids slow down the growth of chondrocytes, lower the production of proteoglycans, and change the way collagen cross-links at the cellular level. This could cause structures to be damaged for longer periods of time [9,10,11,12,13]. Current safety guidelines say that you should limit the number of injections to avoid cumulative toxicity and speed up cartilage loss [12]. People still use CSs a lot because they works well for reducing inflammation and quickly make symptoms go away. They stop the production of leukotrienes and prostaglandins by attaching to the glucocorticoid receptor (GR-α). This stops NF-κB, AP-1, COX-2, and phospholipase A2 [10,11,12]. Their clinical utility is most evident in acute inflammatory exacerbations, where numerous meta-analyses have demonstrated substantial yet transient enhancements typically lasting 2 to 6 weeks [10,11,13]. But using CSs too much can be detrimental for you. Histological studies show that after long-term exposure to CSs, the density of chondrocytes decreases, the synthesis of proteoglycans is impaired, and apoptosis increases [10,11,12,13]. Recent reviews caution that frequent injections may accelerate the degradation of cartilage [10,12]. Clinical guidelines say to be cautious with dosing, only provide CSs during severe flares, and stay away from high-dose combinations of CSs and LAs because they are more toxic to cells [6,12,14].

### 1.6. Key Studies Have Identified the Following Significant Findings

- Meta-analyses show that CSs work the fastest to relieve symptoms, but they do not last as long as HA and PRP [10].
- Guidelines recommend limiting the number of injections to protect the structure and reduce the duration of exposure to the system [12].

Network meta-analyses consistently rank CSs lower than PRP and HA for long-term improvement in pain and function [1,3,4].

Therefore, CSs still have a clear but limited role, especially when it comes to quickly reducing inflammatory surges. However, long-term management strategies are increasingly favoring HA or PRP because they are more durable for both structure and symptoms [1,2,3,4].

#### 1.6.1. Mechanisms and Benefits of Hyaluronic Acid (HA) in Musculoskeletal Care

HA acts through CD44-mediated signaling pathways while restoring synovial viscoelasticity, thereby reducing mechanical stress and inflammatory gene expression. Comparative analyses demonstrate that HA diminishes synovial friction and mechanically stabilizes joint motion, consequently reducing inflammatory microtrauma. Numerous systematic reviews and randomized trials indicate that HA enhances lubrication and shock absorption while mitigating synovial inflammation [9,15,16,17,18,19,20,21]. Signaling pathways that use CD44 change the metabolism of chondrocytes, which is the molecular basis for HA’s protective and anti-inflammatory effects [8]. Physically active groups see the most benefits, which shows HA’s role in restoring joint biomechanics when there is a lot of stress on them [15].

HA has benefits at both the molecular and biomechanical levels. Mechanically, HA restores the viscosity of synovial fluid and enhances boundary lubrication, thereby reducing shear stress on cartilage surfaces. Clinical and biochemical studies examining HA’s molecular weight and viscoelasticity [6,7] support this conclusion. Biochemically, HA interacts with CD44 signaling, inhibiting IL-1β, TNF-α, and MMPs, while enhancing endogenous HA production and chondrocyte viability [9,13,15].

Recent meta-analyses furnish substantial evidence for the intermediate-term efficacy of HA:

- Updated systematic reviews demonstrate that various HA formulations can markedly alleviate pain and enhance functionality [16].
- High-molecular-weight HA appears to have a longer duration of effects, lasting up to six months [9,16].
- Single-injection formulations show small but important improvements over placebo [17].
- HA is most effective for individuals with early to moderate osteoarthritis and for those engaged in sports, where biomechanical restoration is crucial [19].
- Comparative results indicate that HA is better than CSs after 8–12 weeks because it stays in the joint longer and has better viscoelastic properties [9,10,16].
- Several meta-analyses evaluating symptom duration and structural indicators indicate that PRP generally outperforms HA over time [21,22,23].
- Combination therapy with PRP and HA may enhance outcomes by targeting both lubrication and regeneration [23,24,25,26].

In total, HA is best for early to moderate osteoarthritis, where its viscoelastic, anti-inflammatory, and anti-oxidative properties work on the mechanical-biochemical interface of the disease [6,8,9,10,11,12,13,14].

#### 1.6.2. Mechanisms and Benefits of Platelet-Rich Plasma (PRP) in Musculoskeletal Disorders

PRP promotes tissue regeneration through growth factor–driven anabolic cascades, including PDGF-, TGF-β-, and IGF-1–mediated signaling. The anabolic effects of PRP include synovial modulation, with formulations low in leukocytes showing greater effectiveness in reducing synovitis than those high in leukocytes, as shown by systematic reviews and comparative studies [27,28]. Systematic comparisons further show that PRP is better than HA and CS for improving function, especially during follow-up periods of several months or years [21,22,23,24]. Combining PRP and HA is the best way to lubricate and regenerate, and many tests have shown that it works better than PRP alone [23,24,25,26,29]. Molecular studies indicate that regenerative cascades like TGF-β, IGF-1, PDGF, and VEGF are turned on. These cascades enhance chondrocyte viability, promote extracellular matrix deposition, and inhibit inflammatory mediators [30].

PRP is the most promising injectable for regeneration, and increasingly more comparative trials and meta-analyses are showing this. Supraphysiologic concentrations of growth factors, such as PDGF, IGF-1, VEGF, TGF-β, and HGF, are thought to be responsible for its therapeutic potential. These factors work together to help make a new matrix, grow new blood vessels, and change the way inflammation works [30]. Evidence consistently demonstrates that PRP outperforms both CS and HA in achieving long-term enhancement of symptoms and structural integrity:

- Network meta-analyses show that PRP is the best treatment for pain and function over a period of 6 to 12 months [4].
- Meta-analyses confirm PRP’s significant superiority over HA in multiple randomized controlled trials [9,16].
- Mechanistic reviews indicate that PRP can alter inflammatory and anabolic signaling, particularly by reducing IL-1β and TNF-α levels, decreasing MMP-mediated catabolism, enhancing type I and II collagen synthesis, and promoting the health of chondrocytes and tenocytes [31].

These biological effects are why PRP is a better option for regenerative therapy than treatments that only lower inflammation.

Combination therapies of PRP and HA have additive effects. HA viscoelasticity improves lubrication, while PRP’s growth factor profile promotes regeneration. Numerous reviews and clinical trials validate the enhanced efficacy of combination therapy relative to monotherapy [17,20,21].

Standardization is still a big issue. The concentration of platelets, the number of leukocytes, the activation protocols, and the injection methods all vary a lot. Consensus frameworks emphasize the imperative for meticulous documentation of PRP preparation parameters to enhance reproducibility and clinical comparability across studies [32].

#### 1.6.3. Local Anesthetics (LAs)

LA function by reversible blockade of voltage-gated sodium channels but also demonstrate dose-dependent mitochondrial toxicity and chondrocyte apoptosis.

Recent research underscores the disparities in cytotoxicity among various anesthetic agents. Bupivacaine is always the most chondrotoxic of the most common drugs. Numerous mechanistic and toxicity studies have elucidated mitochondrial disruption and caspase-mediated apoptosis as critical pathways by which LAs negatively impact cartilage [6,12,14,33]. These worries are backed up by results from living animals: a single high-concentration intra-articular injection has been shown to cause chondrocyte depletion and matrix degradation in animal models [33]. These results back up guideline advice to limit and use LAs in the joints with care, especially when they are at high concentrations. LAs, which include lidocaine and bupivacaine, are still very important for figuring out what kind of pain you are in and for relieving pain during procedures. The main way they work is by blocking voltage-gated sodium channels in a way that can be undone, which stops nociceptive transmission. Nonetheless, numerous studies underscore substantial dose-dependent chondrotoxicity:

- Hepburn et al. and Gulihar et al. present persuasive evidence that LAs compromise chondrocyte viability and promote matrix degradation, even at clinically prevalent doses [26,27].
- Recent systematic reviews clarify mitochondrial dysfunction, reactive oxygen species production, caspase activation, and extracellular matrix degradation following high-dose LA exposure [14,33,34].
- Animal models validate in vivo joint damage subsequent to intra-articular LA administration with bupivacaine [29].

Consequently, clinical guidelines recommend decreasing concentration and exposure duration, avoiding high-volume intra-articular local anesthetic injections, and limiting their use to diagnostic or peri-procedural settings rather than disease modification [8].

This caution is reinforced by surveys showing that clinicians view LAs, especially bupivacaine, as having a high risk of chondrotoxicity, especially when given at the same time as CS [31].

LAs are still useful for differential diagnosis (for example, locating the source of pain in the joints) and for making procedures more comfortable, but their therapeutic use is limited by strong evidence that they are toxic in a dose-dependent way across cartilage, synovium, and periarticular soft tissues [6,8,12,14,29,31,33].

**Figure 2:**
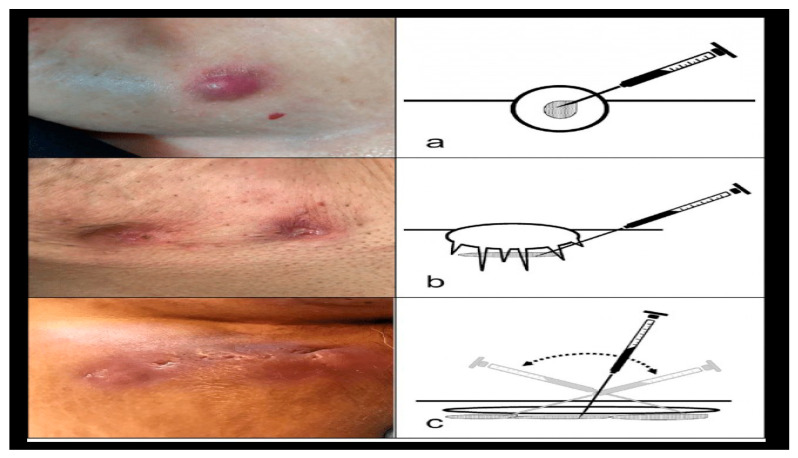
Mechanism-guided clinical selection of injectable therapies based on disease phenotype, biological targets, and expected therapeutic durability. Based on data from Pereira et al. [1], licensed under CC-BY 4.0. (**a**) Corticosteroid injection; (**b**) Hyaluronic acid injection; (**c**) PRP injection.

The mechanism-guided clinical selection of injectable therapies is shown in Figure 2.

Acute inflammatory flares are better treated with CSs; early degenerative disease is better treated with HA; chronic osteoarthritis and tendinopathy are better treated with PRP; and mixed phenotypes may benefit from a combination of PRP and HA therapy.

### 1.7. Clinical Applications

As a review article, this manuscript does not propose original dosing protocols; moreover, dosing regimens vary substantially across studies, limiting standardization. The main characteristics and key findings of representative systematic reviews and meta-analyses included in this review are summarized in Table 1.

Combination treatment strategies are becoming the preferred choice for complex OA phenotypes. Multiple systematic reviews and clinical trials indicate that the combination of PRP and HA yields superior enhancements in pain, function, and structural parameters relative to the use of either agent in isolation [23,24,25,26]. When single-agent therapy is ineffective, guidelines increasingly support multimodal approaches, especially for patients with mixed inflammatory–degenerative phenotypes [8]. Biomarkers are also showing promise for stratification; imaging and molecular markers can help determine people who will respond to PRP and HA, which makes it easier to plan treatment for each person [5].

Comparative effectiveness reviews show which clinical use is best:

- CSs work best when there is a sudden flare-up of inflammation. They quickly relieve symptoms, but the effect does not last long [10,11,12,13].
- HA works best for mild-to-moderate OA. It improves lubrication, biomechanics, and inflammation, and many meta-analyses have shown that the effects last for a long time [6,9,15,17,18,19,20,21].
- PRP is the best treatment for chronic symptomatic OA and tendinopathy because it has functional and structural benefits that last for 6 to 12 months or longer [21,22,23,24,25,26].
- LAs can damage cartilage in high doses and do not change the disease’s course, so they should only be used for diagnosis or procedures [22,23,24,25,26,27,28,30,32].

More and more people are using combination therapies:

- PRP + HA consistently yields superior outcomes compared to either PRP or HA alone, enhancing both lubrication and regeneration, as demonstrated by systematic reviews and randomized trials [23,24,25,26].

Guidelines are increasingly calling for multimodal, mechanism-targeted methods for dealing with complex phenotypes. These methods use molecular signatures, imaging results, and clinical response patterns [33,34]. Imaging and biomarker studies demonstrating improved structural parameters post-PRP and HA, such as increased cartilage thickness and reduced synovial inflammation, further substantiate the translational significance. People do not usually see these changes after receiving corticosteroid shots [5].

Combining molecular mechanisms with disease phenotype makes it easier to make decisions in the clinic:

#### 1.7.1. Best Use Time for Agents and Main Benefits

CSs are used for acute inflammatory flares as they provide the quickest relief. HAOA, which is used for mild to moderate conditions, restores viscoelasticity. PRP is used for chronic osteoarthritis and tendinopathy, providing long-term regenerative effects.

#### 1.7.2. LA for Diagnostics and Peri-Procedural Use: Immediate, Rapid Analgesia

Combination therapies, especially PRP + HA protocols, yield superior results in patients exhibiting mixed biomechanical and inflammatory pathologies [23,26,34]. CSs work quickly to make symptoms better, but they do not last long and can hurt cartilage if you take them too often. HA helps with biomechanics, but only for a short time. PRP, on the other hand, helps the body heal itself, which gives it functional and structural benefits that last longer. LAs are still useful for diagnosis and procedures, but they can hurt cartilage if you take too much.

### 1.8. Safety and Complications

Comprehensive meta-analyses validate the strong tolerability profiles of HA and PRP, with adverse events predominantly manifested as transient local reactions, including post-injection swelling or discomfort. Recent high-quality reviews indicate that both HA and PRP exhibit low complication rates and sufficient safety margins across various patient cohorts [1,6,9,10,11,12,13,14,15,16,17,18,19,20,21]. LAs are still the main concern because they are known to be chondrotoxic in a dose-dependent way. CS, on the other hand, increase the risk of cartilage damage with repeated doses [5,7]. Imaging guidance improves injection accuracy and reduces soft tissue injury, thereby enhancing the safety profile of injectable therapies [28,30].

CSs provide rapid pain relief that endures for about 2–6 weeks, while HA generally shows a delayed effect lasting up to six months. Multiple meta-analyses show that PRP has long-lasting benefits that last longer than six months.

### 1.9. Corticosteroids (CS)

Numerous high-quality trials confirm that CSs continue to be effective in short-term symptomatic exacerbations; however, their structural ramifications remain worrisome. Clinical and mechanistic studies indicate that glucocorticoids stop chondrocytes from growing, lower proteoglycan production, and change the way collagen cross-links [5,6,7,8]. Safety guidelines stress the importance of limiting the number of injections to avoid cumulative toxicity. Older meta-analyses, like Jüni et al. (2015), still agree with what we know today about short-term benefits and the risks that come with them [13].

Risks include cartilage thinning, changes in the subchondral bone, systemic absorption, and faster joint degeneration with repeated injections [5,7].

### 1.10. Hyaluronic Acid (HA)

Comparative studies demonstrate that HA diminishes synovial friction and stabilizes joint movement, consequently alleviating inflammatory microtrauma [6,9,10,11,12,13,14]. CD44-mediated pathways control how chondrocytes use energy, which helps explain how HA protects cells [8].

The greatest benefit goes to groups that are active in sports, which shows how HA helps joints work better when they are under a lot of stress [14].

The safety profile of HA is very good overall. Adverse events are rare and are not usually serious. It is possible to have pseudosepta reactions, but they do not happen very often [9,10,11,12].

### 1.11. Platelet-Rich Plasma (PRP)

PRP has anabolic effects, including synovial modulation. Formulations with fewer leukocytes are more effective at reducing synovitis than those with more leukocytes [18,19].

Systematic reviews underscore the efficacy of PRP in functional enhancement, while combination formulations of PRP and HA enhance both lubrication and regenerative processes [16,17,20,21]. Molecular studies have documented the activation of regenerative cascades, including TGF-β and IGF-1 [29].

PRP is autologous, which means that it has an excellent safety profile.

The most common reaction is a temporary inflammatory flare after the injection, and serious side effects are very rare [9,15,16,17,18,19,20,21].

### 1.12. Local Anesthetics

Recent studies indicate substantial differences in cytotoxicity among LAs, with bupivacaine demonstrating the highest chondrotoxic potential. Mechanistic studies clarify mitochondrial dysfunction, oxidative stress, and caspase-mediated apoptosis following exposure to LA [22,23,24,26,27].

In vivo studies confirm that a solitary high-concentration injection can cause quantifiable cartilage damage [32].

As for the safety profile of LAs, there is strong evidence for dose-dependent chondrotoxicity. Bupivacaine is more toxic than lidocaine. Using imaging guidance (like ultrasound or fluoroscopy) makes procedures safer and lowers the risk of soft tissue damage for all injectables [28].

#### 1.12.1. Translational Significance

Testa et al. elucidated the molecular mechanisms through which CS, HA, and PRP exert their biological effects, demonstrating distinct pathway-specific actions for each injectable agent [30]. The data shows that PRP only boosts the activity of genes that help fix damage, while HA mostly affects inflammation and how joints move. CS quickly stop inflammatory pathways, but only for a short time [19]. Precision medicine frameworks demonstrate that integrating biomarkers, imaging findings, and molecular signatures can support individualized injectable therapy selection [6].

A mechanistic continuum includes interactions at the receptor level, such as activating the glucocorticoid receptor for CS, engaging CD44 for HA, signaling cascades for platelet-derived growth factor and TGF-β for PRP, and blocking sodium channels for lidocaine. These interactions have downstream effects on pain modulation, inflammation, tissue quality, and patient-reported outcomes [6,7,8].

Recent biomarker studies strengthen this translational link. Imaging-based predictors, including synovial hypertrophy, bone marrow lesions, and cartilage composition metrics (such as T2 mapping and dGEMRIC), have shown promise in identifying individuals who are responsive to HA and PRP, thus enabling the provision of appropriate treatment to the right patients [8]. Molecular investigations corroborate this approach by associating PRP responsiveness with synovial cytokine profiles and HA responsiveness with CD44 expression patterns [31,34].

All of these mechanistic and clinical insights point to the need to move away from choosing injections based on experience and toward biologically informed, personalized musculoskeletal therapies.

#### 1.12.2. Future Directions

Magalon et al. [35] advocate for the global standardization of PRP characterization, emphasizing the need for consistent reporting of platelet concentration, leukocyte content, activation techniques, and injection protocols. This shows how important it is to report on platelet concentration, leukocyte content, activation techniques, and injection protocols in a consistent way [35].

Researchers are also looking into nano-crosslinking and other methods to make HA formulations last longer. Furthermore, they are looking into ways to make corticosteroid preparations with lower doses that are less harmful but still work. Advancements in personalized medicine are expected to facilitate injection selection informed by imaging phenotypes and synovial biomarkers [5].

Modern injection therapy depends on the translational continuum from molecular mechanism to clinical effectiveness. Reviews in *Cartilage* and *Radiology* elucidate the impact of receptor-level interactions, including glucocorticoid receptor activation, CD44 engagement, PRP-related growth factor cascades, and sodium-channel blockade, on biological and clinical outcomes [5,7,31]. These mechanistic insights establish a basis for the development of next-generation injectables tailored to specific pathophysiological profiles.

Biomarker-based strategies are changing quickly:

- Synovial cytokine signatures may be able to tell us how well PRP will work [31].
- Imaging profiles help identify phenotypes that respond to HA, especially in the early stages of cartilage degeneration [8].
- Precision medicine models advocate for the selection of CS for inflammatory phenotypes and PRP for catabolic–degenerative phenotypes, in accordance with emerging pathobiological classifications [6].

The incorporation of mechanism-guided therapy selection corresponds with global movements towards individualized musculoskeletal treatment and is anticipated to propel forthcoming advancements in soft tissue and intra-articular injectables.

## 2. Conclusions

A mechanism-guided interpretation suggests that injectable therapies should be selected based on dominant inflammatory, degenerative, or regenerative disease phenotypes rather than symptom severity alone. CSs provide quick relief from symptoms because they have strong anti-inflammatory effects, but they do not help in the long term. On the other hand, HA helps in the short term by restoring the elasticity of joints and controlling inflammation. PRP has the best chance of long-term functional improvement and tissue modulation through growth factor–mediated regenerative pathways. These results highlight that injectable therapies are not interchangeable and should be chosen based on the predominant biological and structural characteristics of the disease rather than solely on symptom severity.

From a clinical perspective, these findings support a shift from using the same injection algorithms for everyone to using treatment strategies that are based on mechanisms and phenotypes. Aligning injectable selection with inflammatory, degenerative, or regenerative disease profiles can improve treatment effectiveness while reducing unnecessary exposure and risk. Combination therapies, particularly the integration of PRP with hyaluronic acid, may yield superior advantages in mixed phenotypes by addressing both biomechanical and biological determinants of disease. Doctors should think about imaging guidance, safety issues, and things that are unique to each patient when they make decisions. This will help them achieve the best results and keep joints healthy for a long time.

To improve personalized injection strategies, future studies should focus on standardizing PRP characterization, using biomarkers to group patients, and using imaging to measure outcomes. We need well-designed comparative trials that use molecular biomarkers, advanced imaging, and longitudinal structural endpoints to better define responder profiles, optimize treatment sequencing, and close the gap between biological mechanisms and clinical practice. Moving injection therapy along this precision-guided continuum will be very important for obtaining better long-term results in treating musculoskeletal problems.

## Figures and Tables

**Table 1:** Characteristics of representative review and meta-analysis studies included in this review.

Author (Year)	Injectable Agent(s)	Study Type	Condition	No. of Included Studies	Main Outcomes Assessed	Key Conclusions
Pereira et al. (2025) [1]	CSs, HA, PRP	Systematic review and network meta-analysis	Knee osteoarthritis	79 RCTs	Pain, function, safety	PRP ranked highest for long-term outcomes; effects of CSs were short-lived; HA had intermediate efficacy.
Jones et al. (2018) [2]	CSs, HA, PRP	Narrative review	Knee osteoarthritis	NR	Clinical efficacy, mechanisms	Injectables should be selected according to OA phenotype.
Bensa et al. (2025) [3]	CSs, HA, PRP	Narrative review	Knee osteoarthritis	NR	Pain, function, durability	Biological injectables show superior durability vs. CSs.
Gupta et al. (2025) [4]	CSs, HA, PRP	Bayesian network meta-analysis	Knee osteoarthritis	35 RCTs	Pain, function	PRP demonstrated the most sustained benefit.
Bruyère et al. (2025) [16]	HA	Systematic review	Knee osteoarthritis	24 RCTs	Pain, function	HA significantly improves pain and function in early–moderate OA.
Yi et al. (2025) [17]	PRP	Overview of reviews	Knee osteoarthritis	15 reviews	Pain, function	PRP is consistently superior to placebo, HA, and CSs.
Yi et al. (2025) [17]	PRP + HA	Systematic review and meta-analysis	Knee osteoarthritis	10 RCTs	Pain, function, safety	Combination PRP + HA is superior to PRP alone.
Pirri et al. (2024) [28]	Local anesthetics	Narrative review	Musculoskeletal injections	NR	Chondrotoxicity, safety	Dose-dependent cytotoxicity; bupivacaine had the highest risk.
Gulihar et al. (2015) [27]	Local anesthetics	Systematic review	Articular cartilage	41 studies	Chondrocyte viability	Strong evidence of chondrotoxicity from local anesthetics.

## Data Availability

All data supporting the findings are included within the article.
